# Three species of land leeches from Taiwan, *Haemadipsa rjukjuana* comb. n., a new record for *Haemadipsa picta* Moore, and an updated description of *Tritetrabdella taiwana* (Oka)

**DOI:** 10.3897/zookeys.139.1711

**Published:** 2011-10-25

**Authors:** Yi-Te Lai, Takafumi Nakano, Jiun-Hong Chen

**Affiliations:** 1 Institute of Zoology, National Taiwan University, No. 1, Roosevelt Road, Section 4, Taipei 106, Taiwan; 2 Department of Biology, University of Eastern Finland, P. O. Box 111, FI 80101 Joensuu, Finland; 3 Laboratory of Systematic Zoology, Department of Zoology, Graduate school of Science, Kyoto University, Kitashirakawa-Oiwakecho, Sakyo-ku, Kyoto 606-8502, Japan

**Keywords:** Land leech, *Haemadipsa rjukjuana*, *Haemadipsa picta*, *Tritetrabdella taiwana*, *Haemadipsa japonica*, Taxonomy, Taiwan

## Abstract

Three species of land leeches, including a new combination *Haemadipsa rjukjuana* **comb. n.**, a new record for *Haemadipsa picta* Moore, as well as an updated description for *Tritetrabdella taiwana* (Oka), are reported in this study. Morphological characters and DNA barcode analysis were used to identify these species. In addition, since *Haemadipsa rjukjuana* had been regarded as a variety of the Japanese land leech *Haemadipsa japonica* for a century, morphological differences between these two species were also compared.

## Introduction

Land leeches are generally referred to a group of sanguivorous species belonging to different genera that mainly live in the Indo-Pacific. These species are adapted to terrestrial life, but are restricted to damp forests with high humidity; hence, the majority of species are distributed in tropical and subtropical areas (Sawyer 1986). Because of their bloodfeeding habit, land leeches have been observed and collected in damp forests during surveys, and many of these species have been described in the last century. According to Sket and Trontelj (2008), there are about 60 described land leech species, of which 50 belong to the Family Haemadipsidae, while the rest are in the Family Xerobdellidae. However, the taxonomy of these bloodfeeding land leeches remained complicated and ambiguous for decades prior to the advent of molecular phylogenetic research at the end of the last century. Sawyer (1986) suggested that the single family Haemadipsidae, which comprises a total of 17 genera divided into the duognathous (two jaws) and trignathous (three jaws) series, should include all the land leech species. However, Moore (1946), (Ringuelet (1953, 1972, 1982) and (Richardson (1975, 1978) suggested dividing about 30 genera into several families (including Haemadipsidae), to distinguish between bloodfeeding land leeches from the Indo-Pacific, which only have two jaws, and other regions. Ultimately, the controversial higher-level taxonomy of bloodfeeding land leeches was resolved through a series of molecular phylogenetic studies (Trontelj et al. 1999; Borda and Siddall 2004; Kutschera et al. 2007; Borda et al. 2008). Based on these studies, bloodfeeding land leeches are separated into two families, Haemadipsidae and Xerobdellidae, using both morphological characteristics and molecular analysis. However, despite recently published conclusions about the higher taxonomic level of bloodfeeding land leeches, the species-level taxonomy of these groups has been largely ignored. Therefore, a land leech species, such as *Haemadipsa zeylanica* Whitman, for which many subspecies or varieties have been described, has not been critically investigated.

DNA barcoding is a system of species identification that uses DNA sequences (Hebert et al. 2003). Mitochondrial cytochrome *c* oxidase subunit 1 (COI) sequences have been selected as the DNA barcode for most animal phyla (Hebert et al. 2003). Recently, the need for DNA barcoding in the species identification of leeches has been widely discussed (Bely and Weisblat 2006; Siddall et al. 2007). Bely and Weisblat (2006) found that the laboratory isolates of *Helobdella* used for developmental biological studies, which had previously been identified as *Haemadipsa triserialis* Blanchard and *Haemadipsa robusta* Shankland et al., represented five distinct species, including at least two cryptic species. By using the same DNA technique, Siddall and Budinoff (2005) identified the widespread introduction of *Helobdella europaea* Kutschera into Australia, New Zealand, South Africa, and Hawaii. In Taiwan, Lai et al. (2009) also identified *Haemadipsa europaea* through DNA barcoding analysis, as well as two new *Helobdella* species, *Haemadipsa octatestisaca* Lai and Chang and *Haemadipsa melananus* Lai and Chang. However, the DNA barcoding of bloodfeeding leeches has only been used to identify different species of commercially available medical leeches, in addition to resolving the taxonomy of these leeches (Trontelj and Utevsky 2005; Siddall et al. 2007), rather than for taxonomic studies of bloodfeeding land leeches.

This study presents the first case where DNA barcoding is applied to assist with new species descriptions for bloodfeeding land leeches. Here, we present a report on two *Haemadipsa* leeches, including a new combination, which was regarded as a variety of *Haemadipsa japonica* for a century, and a new record of a species in Taiwan. In this study, we compare this new combination against *Haemadipsa japonica*. In addition, we also include descriptions and molecular analysis of a rarely described haemadipsoid species, *Tritetrabdella taiwana* (Oka).

## Methods

### Sample collection and preservation

From 2001 to 2009, we collected leeches in the suburban hills and mountains around Taiwan. Collection strategies involved walking along forest trails and streams, as well as searching through damp undergrowth, to attract leeches. We also received leech specimens collected by other field surveyors and friends. Specimens were anesthetized and relaxed in 10% ethanol solution, followed by fixation in 10% formalin solution for 24 h. The specimens were then preserved in 70% ethanol solution for morphological inspection and dissection, or were directly preserved in 75% ethanol solution for DNA extraction and barcoding analyses. All specimens were deposited in the Invertebrate Zoology and Cell Biology Lab, Department of Life Science in National Taiwan University, Taipei, Taiwan.

### DNA extraction, PCR, and DNA sequencing

Tissue from the caudal sucker was used to minimize the possibility of contamination from host/prey DNA found in gastric and intestinal regions. The Genomic DNA Mini Kit (Tissue) (IBI Scientific, Iowa, USA) was used for tissue lysis and DNA puriﬁcation. The extracted DNA was stored at -20 °C.

A 658 bp mitochondrial cytochrome *c* oxidase subunit I (COI) DNA fragment was amplified using the universal primers LCO1490 (5’-GGT CAA CAA ATC ATA AAG ATA TTG G-3’) and HCO2198 (5’-TAA ACT TCA GGG TGA CCA AAA AAT CA-3’) (Folmer et al. 1994). PCR amplifications were carried out in a 50 μl total volume using 1 cycle at 94 °C for 1 min, followed by 6 cycles of denaturation for 30 s at 94 °C, annealing for 30 s at 45 °C, and extension for 50 s at 72 °C, and later by 35 cycles of denaturation for 30 s at 94 °C, annealing for 30 s at 54 °C, and extension for 50 s at 72 °C, with a final extension at 72 °C for 10 min.

The PCR products were checked using 1.0% agarose gel electrophoresis and sequenced in both directions using the same primers as in the PCR. Sequencing was performed with the ABI PRISM BigDye Terminator Cycle Sequencing Ready Reaction Kit, V3.1 (Applied Biosystems, CA, USA). Products were analysed with a ABI 3730 XL DNA Analyzer (Applied Biosystems).

### DNA barcoding analyses

COI sequences of haemadipsoid leeches reported by Siddall and Burreson (1998), Borda and Siddall (2004), and Borda et al. (2008) were retrieved from GenBank. Sequences of xerobdellid leeches reported by Borda and Siddall (2004) and Borda et al. (2008) were also retrieved from GenBank and used as outgroups. All sequences used were aligned using the default settings of Clustal X 1.81 (Thompson et al. 1997). A homologous fragment of 604 bp of the COI sequence was used in this study. The sequences obtained were submitted to GenBank (Table 1). Phylogenetic analyses were conducted using MEGA 3.1 (Kumar et al. 2004). Neighbor joining (NJ) analyses were performed using Kimura’s two–parameter model (Kimura 1980). A bootstrap analysis with 1,000 pseudo–replicates was conducted to evaluate the robustness of the clades.

**Table 1.** Collection localities and GenBank accession numbers of haemadipsoid leeches used in the phylogenetic analyses.

Taxon	Locality	GenBank accession No.	Reference
*Ingroup*			
*Chtonobdella bilineata*	Australia	AF003267	Siddall and Burreson (1998)
*Chtonobdella whitmani*	Australia	EU100087	Borda et al. (2008)
*Haemadipsa interrupta*	Thailand	EU100091	Borda et al. (2008)
*Haemadipsa hainana* L00153A	Hainan Island, China	HQ322473	This study
*Haemadipsa japonica*	Japan		Borda and Siddall (2011)
*Haemadipsa picta*	Borneo	AY425445	Borda and Siddall (2004)
*Haemadipsa picta* L00151A	Taiwan	HQ322470	This study
*Haemadipsa picta* L00100A	Taiwan	HQ322471	This study
*Haemadipsa picta* L00152A	Taiwan	HQ322472	This study
*Haemadipsa rjukjuana* L00112A	Taiwan	HQ322438	This study
*Haemadipsa rjukjuana* L00111A	Taiwan	HQ322439	This study
*Haemadipsa rjukjuana* L00110A	Taiwan	HQ322440	This study
*Haemadipsa rjukjuana* L00114A	Taiwan	HQ322441	This study
*Haemadipsa rjukjuana* L00113A	Taiwan	HQ322442	This study
*Haemadipsa rjukjuana* L00115A	Taiwan	HQ322443	This study
*Haemadipsa rjukjuana* L00116A	Taiwan	HQ322444	This study
*Haemadipsa rjukjuana* L00117A	Taiwan	HQ322445	This study
*Haemadipsa rjukjuana* L00118A	Taiwan	HQ322446	This study
*Haemadipsa rjukjuana* L00119A	Taiwan	HQ322447	This study
*Haemadipsa rjukjuana* L00120A	Taiwan	HQ322448	This study
*Haemadipsa rjukjuana* L00121A	Taiwan	HQ322449	This study
*Haemadipsa rjukjuana* L00122A	Taiwan	HQ322450	This study
*Haemadipsa rjukjuana* L00123A	Taiwan	HQ322451	This study
*Haemadipsa rjukjuana* L00125A	Taiwan	HQ322452	This study
*Haemadipsa rjukjuana* L00126A	Taiwan	HQ322453	This study
*Haemadipsa rjukjuana* L00127A	Taiwan	HQ322454	This study
*Haemadipsa rjukjuana* L00129A	Taiwan	HQ322455	This study
*Haemadipsa rjukjuana* L00131A	Taiwan	HQ322456	This study
*Haemadipsa rjukjuana* L00132A	Taiwan	HQ322457	This study
*Haemadipsa rjukjuana* L00133A	Taiwan	HQ322458	This study
*Haemadipsa rjukjuana* L00135A	Taiwan	HQ322459	This study
*Haemadipsa rjukjuana* L00136A	Taiwan	HQ322460	This study
*Haemadipsa rjukjuana* L00138A	Taiwan	HQ322461	This study
*Haemadipsa rjukjuana* L00098A	Ryukyu Islands, Japan	HQ322462	This study
*Haemadipsa sumatrana*	Borneo	AY425446	Borda and Siddall (2004)
*Haemadipsa sylvestris*	Vietnam	AF003266	Siddall and Burreson (1998)
*Idiobdella seychellensis*	Seychelle Islands	EU100094	Borda et al. (2008)
*Malagabdella fallax*	Madagascar	EU100096	Borda et al. (2008)
*Nesophilaemon skottsbergii*	Juan Fernandez Islands	EU100098	Borda et al. (2008)
*Tritetrabdella taiwana* L00141A	Taiwan	HQ322463	This study
*Tritetrabdella taiwana* L00142A	Taiwan	HQ322464	This study
*Tritetrabdella taiwana* L00143A	Taiwan	HQ322465	This study
*Tritetrabdella taiwana* L00144A	Taiwan	HQ322466	This study
*Tritetrabdella taiwana* L00146A	Taiwan	HQ322467	This study
*Tritetrabdella taiwana* L00147A	Taiwan	HQ322468	This study
*Tritetrabdella taiwana* L00150A	Taiwan	HQ322469	This study
*Outgroup*			
*Diestecostoma magna*	Mexico	EU100088	Borda et al. (2008)
*Diestecostoma mexicana*	Mexico	EU100089	Borda et al. (2008)
*Diestecostoma trujillensis*	Mexico	EU100090	Borda et al. (2008)
*Mesobdella gemmata* (1)	Chile	AY425454	Borda and Siddall (2004)
*Mesobdella gemmata* (2)	Chile	EU100097	Borda et al. (2008)
*Xerobdella lecomtei*	Slovenia	EU100099	Borda et al. (2008)

## Results

### Species descriptions

#### Haemadipsa rjukjuana

(Oka, 1910) Lai, Nakano & Chen, 2011 comb. n.

http://species-id.net/wiki/Haemadipsa_rjukjuana

Haemadipsa japonica var. *rjukjuana* Oka, 1910. Annot. Zool. Jap. 7: 165–183
Haemadipsa zeylanica Takahashi, 1934. Rep. Jpn. Sci. Assoc. 10: 744–749
Haemadipsa zeylanica var. Moore, 1938. B. Raffles. Mus. 14: 64–80
Haemadipsa japonica var. *rjukjuana* Keegan & Toshioka, 1968. Biomed. Rep. 406 Med. Lab. No. 16. United States Army Medical Commend, Japan.
Haemadipsa zeylanica Wu, 1979. Quart. J. Taiwan Mus. 32: 193–207
Haemadipsa japonica Yang, 1996. Fauna Sinica, Annelida: Hirudinea. Science Press, Beijing, China.
Haemadipsa japonica var. *ryukyuana* Lai & Chen, 2005. Note Newsl. Wildlifers 9: 10–14
Haemadipsa japonica var. *ryukyuana* Lai et al., 2009. Zootaxa 2068: 27–46

##### Material examined.

L00062 & L00063 collected at 21^st^ Sept. 2003 in the mountain in Yilan County.; L00064 collected at 16^th^ Mar. 2002 in the Fushan Botanical Garden in Yilan County; L00101 collected at 23^rd^ Apr. 2005 in the Fushan Botanical Garden in Yilan County; L00102 collected at 20^th^ Jan. 2002 in the Fushan Botanical Garden in Yilan County; L00103 collected at 21^st^ May 2005 in the Fushan Botanical Garden in Yilan County. L00026 collected at 20^th^ Jan. 2002 in the Fushan Botanical Garden in Yilan County; L00027 collected at 19^th^ Feb. 2002 in the Fushan Botanical Garden in Yilan County; L00098A (two specimens) collected at 16^th^ Mar. 2009 in Mt. Otake, Akuseki-jima, Tokara Islands, Japan (29°27'56"N, 129°35'40"E); L00104 (three specimens) collected at 30^th^ May 2005 in Wufong Town, Hsinchu County; L00105 collected at 16^th^ Mar. 2002 in the Fushan Botanical Garden in Yilan County; L00106 collected at 20^th^ Jan. 2002 in the Fushan Botanical Garden in Yilan County; L00107 collected at 27^th^ Mar. 2004 in Hsoulin Town, Hualien County; and L00108 (two specimens) collected at 4^th^ Aug. 2004 in the mountain in Yilan County.

##### Diagnosis.

**Figure 1.**
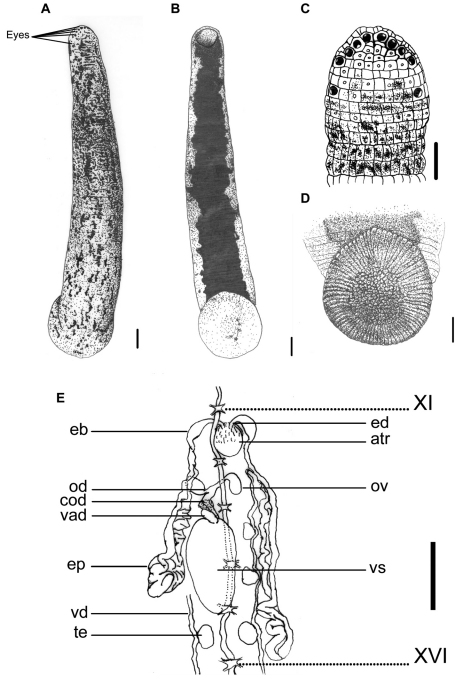
*Haemadipsa rjukjuana*. **A** Dorsum. **B** Venter. **C** Dorsal head. **D** Venter of caudal sucker. **E** Reproductive system. atr. Atrium; cod. Common oviduct; eb. Ejaculatory bulb; ep. Epididymis; od. Oviduct; ov. Ovary; te. Testisacs; vad. Vaginal duct; vd. Vas deferens; vs. Vaginal sac. XI and XVI indicate the orders of the ganglia. Each scale indicates 1 mm in the Figure respectively.

This species can be recognized by the reddish, yellowish, or grayish brown dorsum that is blotched with elongated irregular black spots that are more or less connected, and the absence of a distinct median stripe (Fig. 1A). The nearly solid black venter with irregular margins clearly distinguishes this species from other land leech species (Fig. 1B).

##### External characters.

Body length 14–37 mm, maximum body width 2.5–5.3 mm, up to 10.5 mm in specimen filled with blood; anterior sucker diameter 1.2–2.4 mm, posterior sucker diameter 2.6–5.6 mm. Body elongated, slenderly cylindrical, with dorsum moderately depressed from the end of body to the head; venter more or less flat in relaxed specimens. Head of dorsal anterior sucker with usual sub-triangular outline (Fig. 1C), venter of lip with the broad median field marked by narrow, longitudinal ridges and a deep median fissure. Anterior sucker deep, wide, triangularly cupuliform with well-developed lateral buccal lobes and frill. Posterior sucker nearly circular, slightly longer than wide, diameter equal to or a little larger than maximum body width, with a definite anterior median prominence but no sharply hooked papilla. Auricles large, white or even translucent, trilobate with the middle lobe smallest, and conspicuous by their color in contrast with the body color.

Dorsum strongly tessellated, with three pairs of paramedian, intermediate and supramarginal lines of prominently elevated, translucent-tipped sensillae, and also scattered areas bearing smaller semi-transparent tipped sensillae on annuli in addition to the sensory one. Venter tessellated less and more smooth than dorsum, with white or translucent tipped sensillae in arrangement as those on the dorsum. Dorsum of posterior sucker tessellated, with five or six irregular circles of polygonal areas. Venter of posterior sucker with rays 71 or 72, with strongly flattened ridges terminated in little rounded lobes at the margin, and not penetrated into the relatively large central areolated region (Fig. 1D).

When alive, dorsum reddish, yellowish, or grayish brown, with scattered elongated, more or less connected lateral-posteriorly, irregular black spots. No distinct median stripe on the dorsum, but in some specimens the mid-dorsum less blotched by spots, sometimes similar to an indistinct pale mid-dorsal stripe (Fig. 1A). In lateral body, the region around the sensillae lacking in spots, sometimes similar as a broken pale lateral stripe. Venter uniform, solid black, with highly irregular lateral margins which usually connected with the irregular spots from the lateral body (Fig. 1B). Dorsum of posterior sucker the same but more or less brighter in color than dorsum, with scattered black spots (Fig. 1A). Venter of posterior sucker fawn, sometimes with few scattered dark spots (Fig. 1D).

Eyes five pairs, punctiform, arranged respectively at II (2^nd^ annulus), III (3^rd^ annulus), IV (4^th^ annulus), V (5^th^ annulus) and VI (8^th^ annulus) in parabolic arc (Fig. 1C).

Ninety-seven annuli in total. I, II and III uniannulate, with irregular areas divided and with sensillae in the interocular region. IV uniannulate and the interocular region being divided into irregular areas with sensillae in two transverse rows. V biannulate dorsally ((a1a2)>a3) and uniannulate ventrally, with the a3 as the oral margin of the buccal ring and also the first perfectly definite annulus. VI triannulate with the three annuli approximately equal. VII triannulate with the three annuli of the same length. VIII quadrannulate (a1=a2=b5>b6). IX–XXII midbody somite and quinquannulate, with the five annuli of the same length and a2 projecting above the surface. XXIII quadrannulate (b1=b2=a2>a3). XXIV triannulate (a2>a1=a3). XXV biannulate ((a1a2)=a3), each annulus bearing the first and second auricular lobes at the margins. XXVI uniannulate and bearing the third auricular lobe at the margins. XXVII uniannulate. Anus a small longitudinal slit in XXVII (97^th^ annulus). Gonoporesseparated by five annuli; male at XI b5/b6 (30^th^/31^st^ annulus); female at XII b5/b6 (35^th^/36^th^ annulus); both small transverse slits with pale and projecting margins strictly within furrows.

##### Internal characters.

Jaws three, crescent shaped, moderate size and highly prominent, with 78–80 teeth; one mid-dorsally, the other paired ones ventro-laterally, all in deep buccal chamber beyond the velum. Pharynx in VII–VIII, short, bulbous; with six muscular ridges of spongy wall in which three continuous with the three jaws and the other three intermediately between the formers and surrounded by numerous unicellular salivary glands. Crop in VIII–XIX; with 12 pairs of caeca in VIII–XIX respectively; first nine pairs simple, unlobed, with the first two pairs small and indistinct; while the last pair of caeca in XIX elongated posteriorly to XXIII and lateral to intestine. Intestine in XIX–XXIII, no caeca, ventral to rectum in XXIII. Rectum short, wide, tapered towards anus.

Ten pairs of testisacs at XIII/XIV–XXII/XXIII. Vas deferens enters epididymis in XII/XIII or XIII. Epididymis in XII/XIII–XVI, in some cases even to XVIII; asymmetrical, one side of which more massive, located between atrium and vaginal sac, and usually covered the ovisacs and oviducts, while the other side extended posteriorly beyond the vaginal sac, elongated, less massive, and with major part covering on or being covered by the vaginal sac. Ejaculatory bulbs moderately large, elongated ellipsoid, lying at a much lower level by the sides of the atrium, connected by slender ejaculatory ducts with a sharp turning backwards into atrium in XI. Atrium large, rounded, conspicuous, rising well dorsad of the level of the nerve cord passing along in the right side. Prostate glands a layer of loosely compact. Ovisacs in XII, large, connected with long and curled common oviduct. Vaginal sac in XIV–XVI, cephalic end sometimes in XIII and the caudal end extended to XVII; elongated egg-shape, bubble-like with thin wall usually, connected with long and thick vaginal stalk extended anteriorly into female gonopore in XII (Fig. 1E).

##### Distribution.

*Haemadipsa rjukjuana* is only recorded in East and South East Asia, including the Indo-Chinese Peninsula, Malay Peninsula, Indonesia, Ryukyu Islands of Japan, and Taiwan. In Taiwan, we recorded this species during recent surveys in the moist forests of low- and middle-elevation mountains in Taipei, Hsinchu, Taichung, Nantou, Pingtung, Yilan, Hualien, and Taitung (Fig. 4).

##### Habitat.

Commonly inhabits the bottom of moist forests. It attaches onto leaf litter, grasses, and low bushes.

##### Host.

Primarily medium- or large-sized mammals, including humans.

##### Remarks.

*Haemadipsa rjukjuana* had previously been recorded with other synonyms, with variable taxonomic status that has rarely been clarified over the last century. Oka (1910) described a new land leech collected from Taiwan, and named it *Haemadipsa japonica* var. *rjukjuana* based on a brief inspection of the external color pattern. After two decades, Takahashi (1934) refered to all the land leeches in Taiwan as *Haemadipsa zeylanica*, which is a variable land leech species widely distributed in South and South-East Asia. Later, Moore (1938) recorded a land leech specimen from the Malay Peninsula, and illustrated both dorsal and ventral color patterns (Fig. 5, Plate IV in Moore 1938). The scattered spots on the dorsum and the solid black venter with irregular lateral margins indicate that it is very similar to the specimens inspected in this study; thus, it could tentatively be confirmed as *Haemadipsa rjukjuana*. However, Moore only recognized it as *Haemadipsa zeylanica* var., despite conspicuous differences in external color patterns, and only provided a few external descriptions, instead of a detailed inspection and investigation on its taxonomic status. In addition, Moore (1938) also mentioned that this variety resembled one of the land leeches illustrated, but not described, by Blanchard (1917). Thirty years later, Keegan et al. (1968) described this variety in more detail. They provided the first description of its reproductive system, and compared it against other varieties of *Haemadipsa zeylanica*. However, in addition to the external color patterns, they stated that there were no differences in the reproductive system between this variety and the subspecies *Haemadipsa zeylanica japonica*, i.e., the species *Haemadipsa japonica* in our study. At the end of the 1970s, Wu (1979) reviewed the previous studies of the leech fauna in Taiwan, and only referred to the land leech species of *Haemadipsa zeylanica* in his list. About two decades later, Yang (1996) mentioned that only *Haemadipsa japonica* was present in Taiwan, as the other common land leech species in Taiwan, *Tritetrabdella taiwana*, which had been described as a new combination by Sawyer (1986), had been mistakenly included. Finally, in the first decade of this century, Lai and his colleborators (Lai and Chen 2005; Lai et al. 2009) stated the uncertain taxonomic status of this variety, and suggested the necessity of further studies. By comparing the morphology of *Haemadipsa japonica* var. *rjukjuana* specimens against *Haemadipsa japonica*, we found significant and consistent differences in both external and internal characteristics (Table 2). Therefore, its taxonomic status should be considered as a new species rather than subspecies or variety.

**Table 2.** Comparison of diagnostic morphological characters between *Haemadipsa rjukjuana* and *Haemadipsa japonica*.

Morphological character	Species	
	Haemadipsa rjukjuana	Haemadipsa japonica
Color pattern, spots and stripes on dorsum	Dorsum reddish, yellowish, or grayish brown, with scattered elongated, more or less connected lateral-posteriorly, irregular black spots, no stripe.	Dorsum red brownish, with a mid-dorsal longitudinal dark stripe and a wide, yellowish mid-dorsal region bordered by two paramedian longitudinal dark stripe, no spot.
Marginal stripe	Sometimes a broken pale stripe formed by a series of pale region around the sensillae.	A continuous, longitudinal pale yellowish stripe.
Color pattern on venter	Uniformly black with highly irregular lateral margins.	Uniformly dark yellowish or red brownish.
Number of transverse rows in interocular region in III	Two	One
Number of rays on venter of posterior sucker	Mostly 71–72	Mostly 74–76
Epididymis morphology and location	Separated, highly asymmetrical. One side more massive, located between atrium and vaginal sac, usually covered the ovisacs and oviducts; while the other side elongated, less massive, usually extended posteriorly beyond the vaginal sac and with major part covering on or being covered by the vaginal sac.	Rarely separated, less asymmetrical. Both of the posterior ends highly massive, folded, curled together or extremely close to each other. The main part located between atrium and vaginal sac.

#### Haemadipsa picta

Moore, 1929

http://species-id.net/wiki/Haemadipsa_picta

Haemadipsa picta Moore, 1929.P. Acad. Nat. Sci. Philadelphia 81: 267–295.
Haemadipsa picta Moore, 1935. B. Raffles Mus. 10: 67–78.
Haemadipsa picta Keegan et al., 1968. Biomed. Rep. 406 Med. Lab. No. 16. United States Army Medical Commend, Japan.
Haemadipsa picta Ngamprasertwong et al. 2007.Nat. Hist. J. Chulalongkorn U. 7: 155–159.

##### Material examined.

L00099 collected at 18^th^ Sept. 2005 in Hsoulin Town, Hualien County; L00100A collected at 12^th^ Sept. 2004 in Hualien County; L00151A collected at 15^th^ Oct. 2006 in Hsoulin Town, Hualien County; and L00152A collected at 31^st^ Aug. 2003 in Hualien County.

##### Diagnosis.

**Figure 2.**
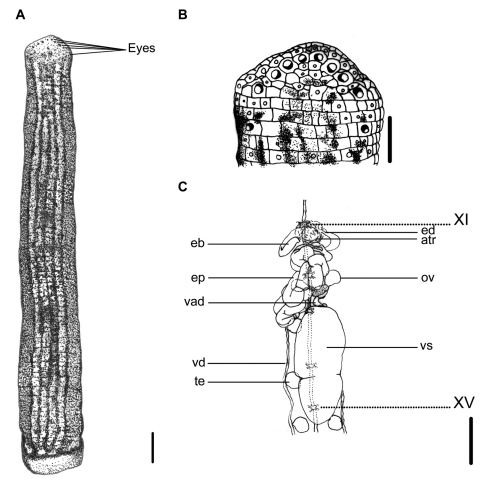
*Haemadipsa picta*. **A** Dorsum. **B** Dorsal head. **C** Reproductive system. atr. Atrium; eb. Ejaculatory bulb; ed. Ejaculatory duct; ep. Epididymis; ov. Ovary; te. Testisacs; vad. Vaginal duct; vd. Vas deferens; vs. Vaginal sac. XI and XV indicate the orders of the ganglia. Each scale indicates 1 mm in the figure respectively.

This species can be recognized by the longitudinally striped color pattern on the reddish brown dorsum, with a broad, bluish-gray, yellow-greenish, or multicolored median-paramedian field that contains three to five black or dark brown broken stripes inside (Fig. 2A). It has a white or pale yellowish longitudinal marginal stripe with dark-spotted borders, as well as a uniformly yellowish brown venter, which distinguishes this species from other land leech species in Taiwan.

##### External morphology.

Body length 13–33 mm, maximum body width 3.0–5.5 mm, anterior sucker diameter 1.3–2.5 mm, posterior sucker diameter 2.5–3.7 mm. Body elongated, slenderly cylindrical, with dorsum moderately depressed from the end of body to the head; venter more or less flat in relaxed specimens. Head of dorsal anterior sucker with usual sub-triangular outline (Fig. 2B); venter of lip with the broad median field marked by narrow, longitudinal ridges and a deep median fissure. Anterior sucker deep, wide, triangularly cupuliform with well-developed lateral buccal lobes and frill. Posterior sucker nearly circular, slightly longer than wide, diameter equal to or a little larger than maximum body width, with a definite anterior median prominence but no sharply hooked papilla. Auricles large, white, trilobate with the middle lobe smallest, and conspicuous by their color in contrast with the body color.

Dorsum strongly tessellated, with areas bearing semi-transparent tipped sensillae in addition to the sensory annuli of each somite. Venter tessellated less and more smooth than dorsum. Dorsum of posterior sucker tessellated, with five or six irregular circles of polygonal areas. Venter of posterior sucker with rays 67 to 72, mostly 71, which in strongly flattened ridges terminating in little rounded lobes at the margin, and not penetrating into the central areolated region.

When alive, body color of reddish brown, or yellow brown in some specimens. Dorsum with three to five longitudinal, black or dark broken stripes of more or less partially and mutually connecting by dark spots in a broad, bluish gray, yellow–greenish, or multicolored median–paramedian field (Fig. 2A). In lateral body, white, pale yellowish, or dusty yellow–greenish marginal stripes bordered by a series of black spots submarginally and supramarginally, especially in half-posterior body. Venter uniform, yellowish brown or resembling color brighter than that in the dorsum, without any spots or stripes. Dorsum of posterior sucker yellow–greenish or yellowish brown, similar to the venter body. Venter of posterior sucker fawn, brighter than venter body.

Eyes five pairs, punctiform, arranging respectively at II (2^nd^ annulus), III (3^rd^ annulus), IV (4^th^ annulus), V (6^th^ annulus) and VI (9^th^ annulus) in parabolic arc (Fig. 2B).

Ninety seven annuli. I, II and III uniannulate, with irregular areas divided and with sensillae in the interocular region. IV biannulate ((a1a2)>a3) and the interocular region being divided into irregular areas with sensillae in two transverse and sometimes oblique rows. V biannulate dorsally ((a1a2)>a3) and uniannulate ventrally, with the a3 as the oral margin of the buccal ring and also the first perfectly definite annulus. VI triannulate (a2>a1>a3). VII triannulate with the three annuli approximately equal. VIII quadrannulate (a1=a2>b5=b6). IX quinquannulate (a2>b1=b2=b5=b6). X–XXII midbody somite and quinquannulate, with the five annuli of the same length and a2 projecting slightly above the surface. XXIII quadrannulate (a2>a1= b5>b6). XXIV triannulate (b1=b2<a2), with b1 & b2 united at the margins and much reduced ventrally, and a2 bearing the first auricular lobe. XXV and XXVI uniannulate, each bearing the second and third auricular lobes at the margins. XXVII uniannulate. Anus in the furrow between XXVII (97^th^ annulus) and the posterior sucker. Gonoporesseparated by five annuli; male at XI b5/b6 (31^st^/32^nd^ annulus); female at XII b5/b6 (36^th^/37^th^ annulus); both moderately large transverse slits strictly within furrows.

##### Internal morphology.

Jaws three, crescent-shaped, small and less prominent, with 78–80 teeth; one mid-dorsally, the other paired ones ventro-laterally, all in deep buccal chamber beyond the velum. Pharynx in VII–VIII, short, bulbous; with spongy muscular walls bearing many radiating fibers and surrounded by numerous unicellular salivary glands; extended into crop in IX. Crop in IX–XIX; with 11 pairs of caeca in each somite respectively; first 10 pairs simple and unlobed, while the tenth pair of caeca in XIX elongated posteriorly to XXIII and lateral to intestine. Intestine in XIX–XXIII, no caeca, with sharp sigmoid flexure and ventral to rectum in XXIII. Rectum short, sharply tapered towards anus.

Ten pairs of testisacs at XIII/XIV–XXII/XXIII. Vas deferens enters epididymis in XIII. Epididymis in XII–XIII, massive, convoluted together, totally posterior to the atrium and covered on a small part of the cephalic end of the vaginal sac. Ejaculatory bulbs of moderate size and form, lying at a low level by the sides of the atrium, and connected by slender ejaculatory ducts to atrium in XI. Atrium large, conspicuous, rising well dorsad of the level of the nerve cord passing along in the left side. Prostate glands a layer of highly compact. Ovisacs in XII/XIII, on which with common oviduct long, sigmoid and slender. Vaginal stalk distinctly shorter than vaginal sac, which of an elongated egg-shape with the small apical end directed caudad in XIV–XVI (Fig. 2C).

##### Distribution.

This species is only recorded in South East Asia, including the Indo-Chinese Peninsula and Borneo. In Taiwan, it is a newly recorded species, and was collected in the moist forests of low- and middle-elevation mountains in Yilan, Hualien and Taitung during our recent surveys (Fig. 4).

##### Habitat.

Commonly found on bushes about 1 m above the ground in moist forests.

##### Host.

Primarily medium- or large-sized mammals, including humans.

##### Remarks.

Unlike many other land leech species which remain on the ground and grass below knee-level, this species usually climbs and waits on bushes and grasses at about 1 m above the ground, and attaches to the hands, arms, shoulders and even neck of passers-by (Keegan et al. 1968). This species has been known to fall onto hikers from higher bushes or leaves (Chun-Chia Huang, pers. comm.). In comparison to other land leech species, the bites of this species are much more painful and difficult-to-heal; thus, *Haemadipsa picta* has been given the common name “stinging land leech” (Moore 1929). Such painful and difficult-to-heal bites were also confirmed by a friend who collected specimens of *Haemadipsa picta* for us (Chun-Chia Huang, pers. comm.). However, this common name should be shared with another similar species, *Haemadipsa ornata* Moore, because it also has a similarly painful bite to *Haemadipsa picta* (Moore, 1929). Nevertheless, this common name is rarely used for *Haemadipsa picta*, while the name, “tiger leech”, has been more commonly used, which refers to the colorful striped pattern (i.e., “picta” in the scientific name).

#### Tritetrabdella taiwana

(Oka, 1910)

http://species-id.net/wiki/Tritetrabdella_taiwana

Haemadipsa japonica var. *taiwana* Oka, 1910. Annot. Zool. Jap. 7: 165–183
Haemadipsa zeylanica Takahashi, 1934. Rep. Jpn. Sci. Assoc. 10: 744–749
Haemadipsa japonica var. *taiwana* Keegan et al., 1968. Biomed. Rep. 406 Med. Lab. No. 16. United States Army Medical Commend, Japan.
Haemadipsa japonica var. *taiwana* Wu, 1979. Quart. J. Taiwan Mus. 32: 193–207
Tritetrabdella taiwana Sawyer, 1986. Leech Biol. Behav. Clarendon Press, Oxford, United Kindom.
Haemadipsa japonica Yang, 1996. Fauna Sinica, Annelida: Hirudinea. Science Press, Beijing, China.
Tritetrabdella taiwana Lai and Chen, 2005. Note Newsl. Wildlifers 9: 10–14
Tritetrabdella taiwana Lai et al., 2009. Zootaxa 2068: 27–46

##### Material examined.

L00084 collected at 9^th^ Jun. 2002 in Wulai Town, Taipei County; L00085 collected at 11^th^ Oct. 2003 in Nantou County; L00086 & L00087 collected at 1^st^ Jun. 2004 in Taipei Zoo, Taipei City; and L00109 collected at 15^th^ Feb. 2007 in Taipei City.

##### Diagnosis.

**Figure 3.**
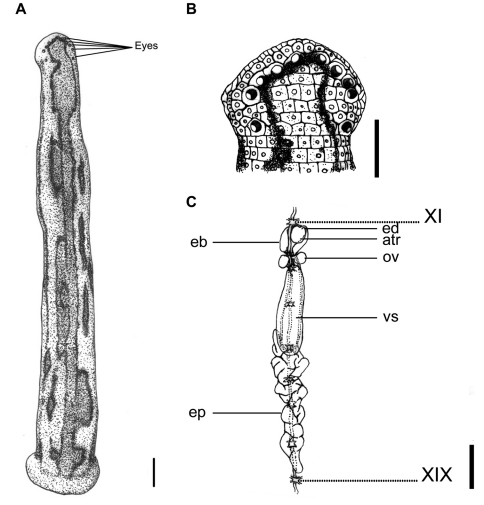
*Tritetrabdella taiwana*. **A** Dorsum. **B** Dorsal head. **C** Reproductive system. atr. Atrium; eb. Ejaculatory bulb; ep. Epididymis; ov. Ovary; vs. Vaginal sac. XI and XIX indicate the orders of the ganglia. Each scale indicates 1 mm in the Figure respectively.

This species can be recognized by the yellowish dorsum with three dark or black bordered brown stripes, in which the supramarginal pair is simple, and the mid-dorsal one has a few irregular, asymmetrical, elongated circles or loops that extend laterally between two stripes. These circles or loops are either connected the mid-dorsal and the supramarginal stripes, or are disconnected from the stripes to form isolated brown spots with a dark border between the two stripes (Fig. 3A). A mid-body somite with four annuli, rather than the usual five annuli of other land leech species in Taiwan, is also an easily recognized characteristic of this species.

##### External morphology.

Body length 12–25 mm, maximum body width 2–4 mm in relaxed specimens and 4–6 mm in specimens filled with blood; anterior sucker diameter 2.0–2.6 mm, posterior sucker diameter 3.0–4.5 mm. Body elongated, slenderly cylindrical, with dorsum depressed moderately from the end to the head; venter flat. Clitellum usually conspicuously wider and thicker. Head of dorsal anterior sucker with broadly rounded, less sub-triangular outline (Fig. 3B); venter of lip soft and finely granular, with no permanent furrows anteriorly but a median fissure posteriorly continuing forward the median velar sinus. Anterior sucker deep, wide, triangularly cupuliform with well-developed lateral buccal lobes and frill. On the sides and floor of the buccal chamber are four pairs of folds or lobes reaching to the membranous velum, through the triangular opening of which the three jaws are visible. Posterior sucker large, broadly ovate, slightly longer than wide, diameter larger than maximum body width, with a definite anterior median prominence but no sharply hooked papilla. Auricles obscure, small, white, and trilobate with the middle lobe smallest.

Dorsum strongly tessellated and areolated, with areas bearing semi-transparent tipped and inconspicuous sensillae on each somite. Venter tessellated less, nearly smooth. Dorsum of posterior sucker tessellated, with four or five irregular circles of polygonal areas. Venter of posterior sucker with rays 57 to 61, not extending into the center and leaving a depressed, faintly tessellated circular central area.

Dorsum yellowish, with three broad, dark or black bordered brown stripes, in which the supramarginal pair simple, and the mid-dorsal one with a few irregular, asymmetrical, elongated circles or loops extending laterally between two stripes. Sometimes these circles or loops either connect the mid-dorsal and the supramarginal stripes, or disconnected from stripes and become isolated brown spots with dark border between two stripes. These stripes differ in exact form and position on each individual. In long preserved specimens, however, color of brown stripes has faded, leaving only longitudinal irregular and asymmetrical black borders on the dorsum (Fig. 3A). Venter uniformly yellowish as the dorsum. Dorsum of posterior sucker yellowish; venter of posterior sucker yellowish, or paler than venter body.

Eyes five pairs, punctiform, large and conspicuous (especially the 1^st^ and 2^nd^ pairs), arranging respectively at II (2^nd^ annulus), III (3^rd^ annulus), IV (4^th^ annulus), V (5^th^ annulus) and VI (8^th^ annulus) in parabolic arc (Fig. 3B).

Eighty-two annuli. I uniannulate, with two rows of areola in which the anterior row much smaller and like those of the ventral face of the lip. II and III uniannulate, with the interocular region being divided into two areas in III. IV uniannulate, with the interocular region being divided into four areas. V biannulate dorsally ((a1a2)>a3) with six interocular areas in the first annulus of this somite in dorsum; uniannulate ventrally as the buccal ring. VI triannulate dorsally (a2>a3>a1) and biannulate ventrally ((a1a2)>a3). VII triannulate (a1=a2<a3). VIII quadrannulate (a1=a2>b5=b6). IX–XXII midbody somite and quadrannulate, with the four annuli of the same length. XXIII triannulate (a1=a2>a3), with a1 & a2 partly united ventrally. XXIV triannulate with the three annuli of the same length. XXV biannulate ((a1a2)>a3), with annuli being divided into irregular polygonal areas, and each annulus bearing the first and second auricular lobes at the margins. XXVI uniannulate, being divided into irregular polygonal areas and with the third auricular lobe at the margins. XXVII uniannulate, being divided into irregular polygonal areas. Anus in XXVII (82^nd^ annulus). Clitellum from X b5 (23^rd^ annulus) to XIII a2 (34^th^ annulus). Gonoporesseparated by three and a half annuli; male at XI b5/b6 (27^th^/28^th^ annulus); female at XII b5 (31^st^ annulus).

##### Internal morphology.

Jaws three, crescent shaped, small and very prominent, with about 45 teeth of the usual form and no salivary papillae. Pharynx in VII–IX, long and wide with spongy wall. Crop in X–XIX; with 10 pairs of caeca in each segment respectively; first nine pairs simple and unlobed, while the last pair of caeca in XIX elongated posteriorly toXXIV and lateral to intestine. Intestine in XIX–XXIV, without caeca,tapered sharply to rectum in XXIV. Rectum large and wide, tapered towards anus in XXVII.

Ten pairs of testisacs at XIII/XIV–XXII/XXIII. Vas deferens enters epididymis in XV and XVI. Epididymis always posterior beyond the vaginal sac, located variably in XV–XVII, in some cases from XIII to XIV with a long tail-like caudal part extending from XIV to XXVIII,; moderately to slightly massive, entangled with each other as a whole mass, and with the anterior part of the mass usually covering on or covered by the vaginal sac. Ejaculatory bulbs large, elongated ellipsoid, lying at about the same level lateral-posteriorly or even totally posteriorly to the atrium, and connected by thick and short ejaculatory ducts to atrium in XI. Atrium moderate or small sized, round, rising dorsad of the level of the nerve cord passing along in the right side. Prostate glands of a thick layer covered on the atrium, ejaculatory duct, and anterior part of the ejaculatory bulbs. Ovisacs in XII, with very short oviduct joined into a short, slender, and curled common oviduct. Vaginal sac located variably in XII–XV, elongated ovate, with a very short vaginal stalk extended ventro-anteriorly into female gonopore in XII (Fig. 3C).

##### Distribution.

**Figure 4.**
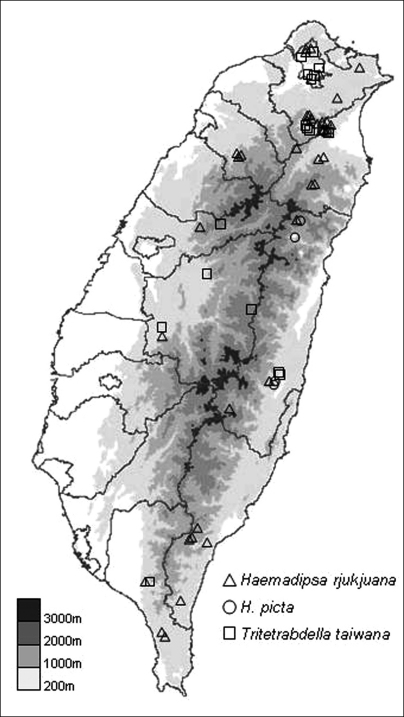
The distribution map of collecting sites for the specimens of the three land leech species collected in recent surveys.

This species is only recorded in East and South East Asia, including the Indo-Chinese Peninsula, Ryukyu Islands of Japan, and Taiwan. In Taiwan, this species is recorded in the moist forests of low- and middle-elevation mountains around the island. In our recent surveys, it was collected in Taipei, Nantou, Pingtung, Yilan, and Hualien (Fig. 4).

##### Habitat.

Commonly found on the ground in moist forests. It attaches to leaf litter, grasses, and bushes on the ground.

##### Host.

Amphibians and medium- or large-sized mammals. The amphibian is probably the primary host, as this species has been frequently recorded parasitizing frogs and toads in Taiwan, including the common toad *Bufo bankorensis* Barbour, the Taipei green tree frog *Rhacophorus taipeianus* Liang & Wang, the temple tree frog *Chirixalus idiootocus* Kuramato & Wang, Swinhoe’s frog *Rana swinhoana* Boulengeer, and the olive frog *Rana adenopleura* Boulengeer.

##### Remarks.

Although Oka (1910) recorded that *Tritetrabdella taiwana* causes a considerable amount of injury by taking blood meals in the nasopharyngeal region of mammals, such as dogs and humans, there is doubt about such parasitic behavior in this species. Oka (1910) mentioned that the leeches of this species enter the nostrils of dogs and men to feed on blood by fastening to the mucous membranes of the nasal passages. However, based on Keegan et al. (1968) and our direct observations of the movement and attaching ability of this species, we argue that the leeches recorded as parasitic in the nasal cavities of mammals may in fact be the nasal leech *Dinobdella ferox* (Blanchard), which is a notorious leech species that specifically parasitizes the nasopharyngeal region of mammal hosts for fast growth before maturation, rather than *Tritetrabdella taiwana*.

In addition, because *Tritetrabdella taiwana* was the only land leech species that has been recorded feeding on frog and toad hosts, sometimes even in groups, it is possible that this species mainly acquires blood from amphibian hosts, whereas mammals covered in body hair are not a primary diet choice. This suggestion may also explain that, while *Tritetrabdella taiwana* is as widely distributed as other land leech species in Taiwan, such as *Haemadipsa rjukjuana*, there are fewer records of *Tritetrabdella taiwana* attacking hikers.

### DNA barcoding analyses

**Figure 5.**
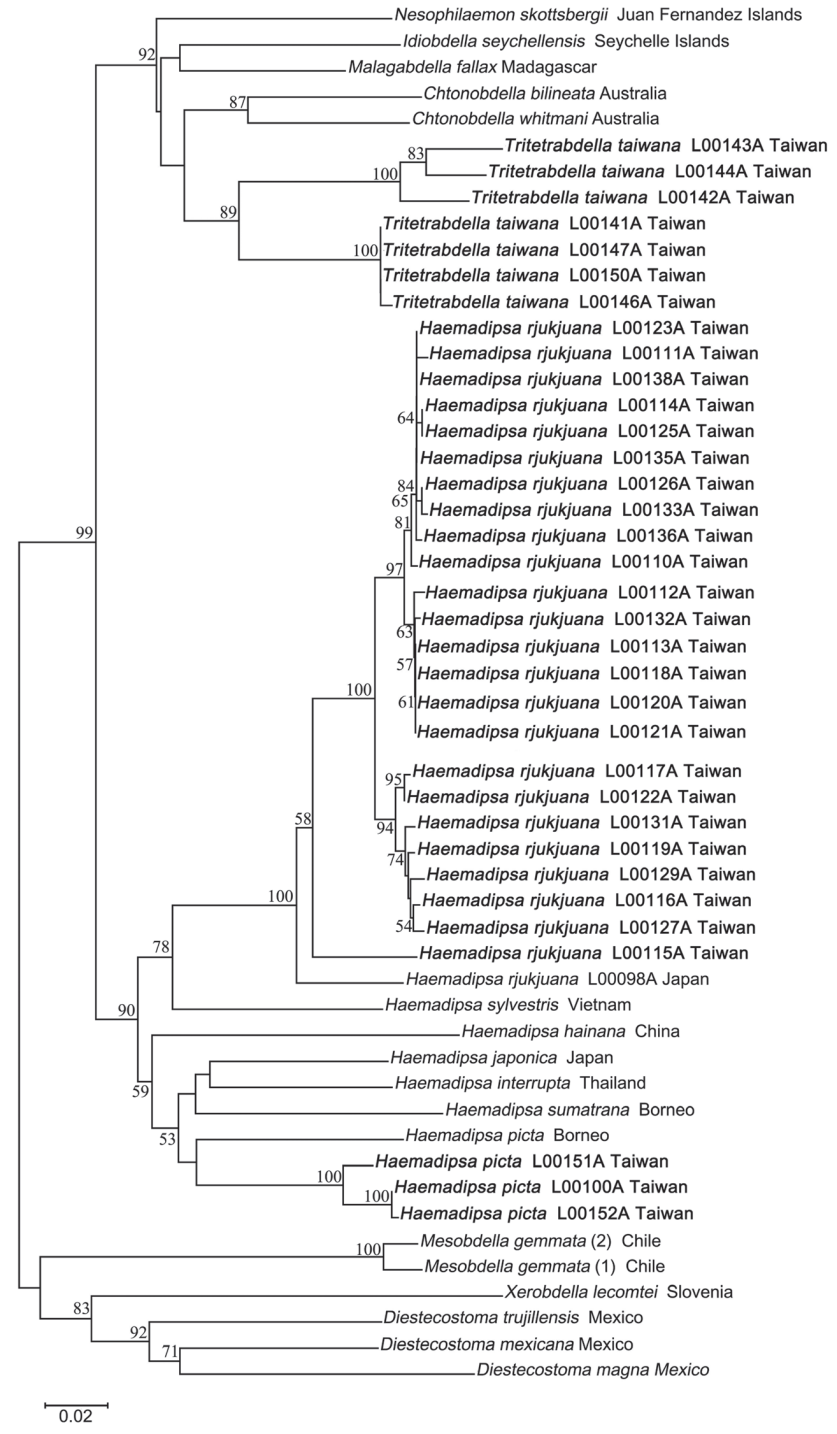
Neighbor joining tree of bloodfeeding land leeches based on COI sequences. Bootstrap values above 50 are shown. Specimens of *Haemadipsa rjukjuana*, *Haemadipsa picta* and *Tritetrabdella taiwana* from Taiwan are marked in bold.

The neighbor-joining tree of haemadipsoid COI genes has high bootstrap support values for the monophyly of each of *Haemadipsa rjukjuana*, *Haemadipsa picta*, *Tritetrabdella taiwana*,and *Mesobdella gemmata* (Fig. 5). The barcoding results also strongly support that *Haemadipsa rjukjuana* is genetically distinct from *Haemadipsa japonica*, with the occurrence of *Haemadipsa picta* in Taiwan also being confirmed. In addition, the phylogenetic relationship of *Tritetrabdella taiwana* as a member of the haemadipsoid leech is also revealed. Our analysis shows that, as a trignathous species, *Tritetrabdella taiwana* is phylogenetically more closely related to duognathous land leech species as opposed to other trignathous species (Fig. 5). This result was also found in a recent study (Borda & Siddall, 2011), in which the authors suggested the establishment of a new subfamily, Tritetrabdellinae, for the newly identified trignathous clade of the genus *Tritetrabdella*.

## Supplementary Material

XML Treatment for Haemadipsa rjukjuana

XML Treatment for Haemadipsa picta

XML Treatment for Tritetrabdella taiwana
